# Immunotherapy of lidocaine allergy by intravenous desensitization using IFN‐gamma from a case: Overcoming impediments using IFN‐gamma during desensitization

**DOI:** 10.1002/ccr3.2038

**Published:** 2019-03-27

**Authors:** Geunwoong Noh, Chul Ki Park, Chang Won Ha

**Affiliations:** ^1^ Department of Allergy, Allergy and Clinical Immunology Center Cheju Halla General Hospital Jeju‐si Korea; ^2^ Department of Dentistry Cheju Halla General Hospital Jeju‐si Korea; ^3^ Department of Pathology Cheju Halla General Hospital Jeju‐si Korea

**Keywords:** desensitization, drug allergy, interferon gamma, lidocaine

## Abstract

Anaphylactic reaction to lidocaine has been reported during a dental procedure. In this trial, a patient who required local anesthesia for dental treatment was desensitized successfully to intravenous lidocaine using IFN‐gamma. Practical general protocols and principles are suggested for the general application of this method for other intravenous drugs.

## 1 INTRODUCTION

The desensitization to intravenous lidocaine using IFN‐gamma as an immunomodulatory adjuvant was successful for 49‐year‐old female patient who required local anesthesia for dental treatment. During the challenge test, the patient immediately exhibited an anaphylactic reaction to 30 ng of intravenous lidocaine. The patient successfully received desensitization treatment for intravenous lidocaine using IFN‐gamma as an immunomodulatory adjuvant following precise and accurate protocols.

Anaphylactic reaction to lidocaine was reported as early as 1957.^1^ Lidocaine‐induced anaphylactic shock during tonsillectomy^2^ and a life‐threatening anaphylactic reaction after the administration of airway topical lidocaine have also been reported.^3^ Local anesthetics (LA) are the most commonly administered drugs in dental practice,^4^ and systemic anaphylaxis following local lidocaine administration during a dental procedure has been reported.^5^

Factors to consider regarding desensitization for drugs are the time requirements and success rates, especially for intravenous drugs. Moreover, a limitation of drug desensitization is that, for patients who failed the desensitization protocol, a new attempt to desensitize was not reported.^6^

IFN‐gamma has been used for the induction of tolerance for food allergies of both the IgE‐mediated and non‐IgE‐mediated types.^7^ IFN‐gamma was reported to have allergen‐specific tolerogenic effects.^8^ IFN‐gamma was introduced for desensitization to aspirin^9^ and, later, cefaclor.^10^

In this case report, IFN‐gamma was introduced as an adjuvant with expected allergen‐specific tolerogenic effects in patients who failed the desensitization protocol, that is, in which patients showed more severe reactions to the same dose repetitively. Successful desensitization to intravenous lidocaine was achieved by the introduction of IFN‐gamma as an immunomodulatory adjuvant for patients who required local anesthesia for dental treatment.

## 2 CASE REPORT

A 49‐year‐old female patient was admitted for desensitization to lidocaine. The patient was to undergo dental treatment for a tooth extraction and implant; however, the patient was allergic to lidocaine. In 2006, the ligament of the patient's right second finger was ruptured, and local anesthesia was administered for the local surgery. The patient exhibited chest tightness, dyspnoea, and resultant respiratory difficulty immediately after a local injection of lidocaine of just several ml. In the same year, the patient received local anesthesia with lidocaine for the removal of a thorn in her left third fingertip. She felt dizziness and exhibited syncope for a short duration.

Thereafter, she received dental treatment with local anesthesia with lidocaine several times without any inconveniences. However, 3 years ago she visited a dental clinic for a tooth implant and again experienced anaphylactic symptoms of dizziness, a short syncope and respiratory difficulty just after receiving a local injection of lidocaine in the gums.

Recently, this patient found out that her teeth should be extracted due to dental caries, and she consulted the Allergy Center, Cheju Halla General Hospital (Jeju‐si, Korea) for the diagnosis and proper treatment of lidocaine allergy. She was admitted for the provocation test to confirm the allergy to lidocaine and to undergo the desensitization to lidocaine.

### 2.1 Laboratory evaluation

Blood and skin prick tests were performed for a general allergy laboratory analysis. In the complete blood count with differential count, the eosinophil fractions were 1.0% (normal range, 0%‐5%) at the initial evaluation and 1.6% just after the desensitization. The initial serum eosinophil cationic protein level was 5.1 μg/L (normal range, 0.0‐14.9 μg/L). The total serum IgE levels were 8.7 KU/L (normal range, 350 KU/L>) at the initial test and 9.2 KU/L just after the desensitization. Specific IgE levels were tested for 40 allergens by MAST (Green Cross^®^, Seoul Korea). Dermatophagoides pteryonyssinus (100 IU/mL <(normal range, 0‐0.34 IU/mL), Grade 6), *Dermatophagoides farinae* (100 IU/mL <, Grade 6), Crab (0.47 IU/mL, Grade 1), and Ox‐eye daisy (0.36 IU/mL, Grade 1) were positive and other allergens (Cat, Dog, Egg white, Milk, Soybean, Shrimp, Peach, Mackerel, Rye, Cockroach, Cladosporium, Aspergillus, Alternaria, Birch/Alder, White oak, Short ragweed, Mug wort, Japanese hop, Hazelnut, Sweet Grass, Bermuda Grass, Cocksfoot, Timothy Grass, Reed, Penicillium, Sycamore, Sallow willow, Cottonwood East, Ash mix, Pine, Japanese Cedar, Acacia, Dandelion, Russian thistle, Goldenrod, and Pigweed) were negative. The skin prick test was negative for 53 tested allergens (*Alternaria alternate*, *Aspergillus fumigatus, Aspergillus niger, Candida albicans*, Clasdosporium, Penicillium Chrysogenum, German cockroach, *Dermatophagoides pteronyssinus, Dermatophagoides farina*, Dog, Cat, Grey Alder(Silver Birch), Grass mix, Mug wort, Short Ragweed, Black willow pollen, Orchard, Bermuda grass, Timothy, English plantain, English Rye grass, Holm oak, Japanese cedar, Cotton flock, Milk, Egg, Chicken, Beef, Pork, Cod, Oyster, Salmon, Prawn, Mackerel, Tuna, Almond, Peanut, Bean, Carrot, Cabbage, Walnut, Maize, Peach, Tomato, Black pepper, Spinach, Wheat, Rabbit, Kapok, Hop, F acacia, Pine, and Poplar).

**Figure 1:**
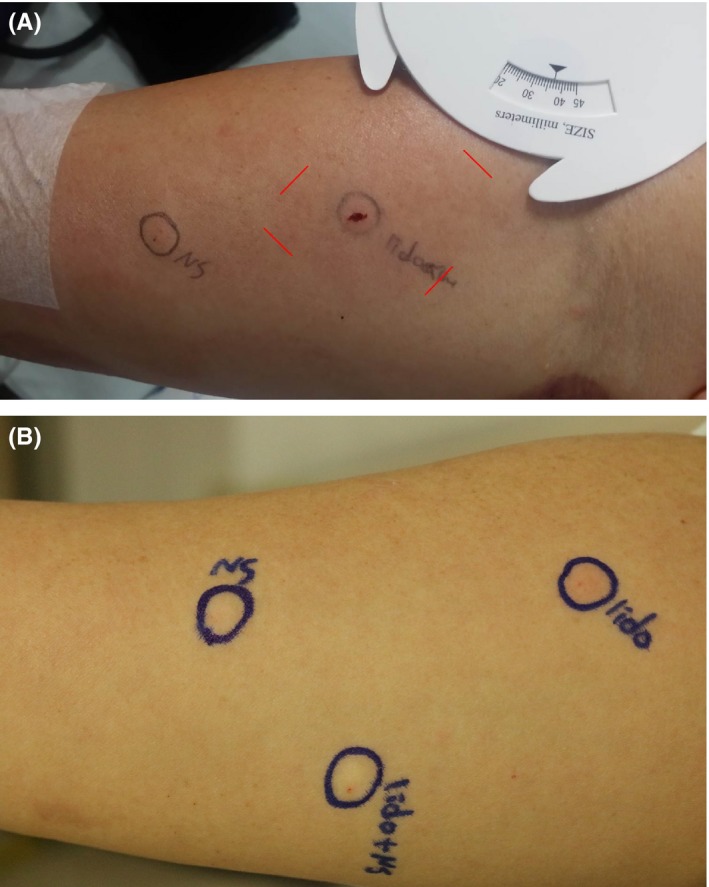
Intradermal test for lidocaine before (A) and after (B) desensitization

The skin prick test for lidocaine was negative. The intradermal test for lidocaine was strongly positive at the initial evaluation and converted to negative just after the desensitization (Figure 1A).

### 2.2 Preparation for intravenous lidocaine challenge/desensitization

Emergency preparations were undertaken for the potential of anaphylactic shock during the challenge test and desensitization. The EKG and percutaneous O_2_ saturation of the patient were monitored continuously during the test. Intravenous steroid, antihistamine, and epinephrine were on hand. Body temperature, blood pressure, and pulse rate were monitored periodically.

### 2.3 Dosing protocol for lidocaine considering the pharmacologic effects, toxic effects, and maximal quantity in dental care

Due to the pharmacologic/toxic effects of lidocaine on the heart, consults were requested from a subdivision of cardiology and the department of internal medicine, and the consults cooperated during the entire process of desensitization, including dosage modulation. In addition, for the original purpose of dental care, a consultation was requested from the department of dentistry to determine the maximal use of lidocaine during the dental procedure. Due to the patient's psychologic problems resulting from her experiences of anaphylaxis, a consultation was requested from the department of psychiatry, and the patient was supported psychologically during the entire process of desensitization and final dental care.

### 2.4 The use of IFN‐gamma for lidocaine desensitization to overcome the impediment

The patient was to receive local anesthesia the dental care, and there was no other modality for desensitization to lidocaine. For the new advanced concept in the case of impediment during desensitization at a certain dose, IFN‐gamma was expected to have allergen‐specific tolerogenic effects similar to those observed in food allergies in situations where desensitization meets an impediment and fails. For the application of IFN‐gamma in the desensitization of drug allergy, a consultation was requested from the Clinical Pharmacy Coordinator/Antimicrobial Stewardship Pharmacist, Central Valley Specialty Hospital (Modesto, CA) concerning the immunologic effects of IFN‐gamma for drug desensitization.

### 2.5 Principle of desensitization using IFN‐gamma

#### 2.5.1 Dosage

**Figure 2:**
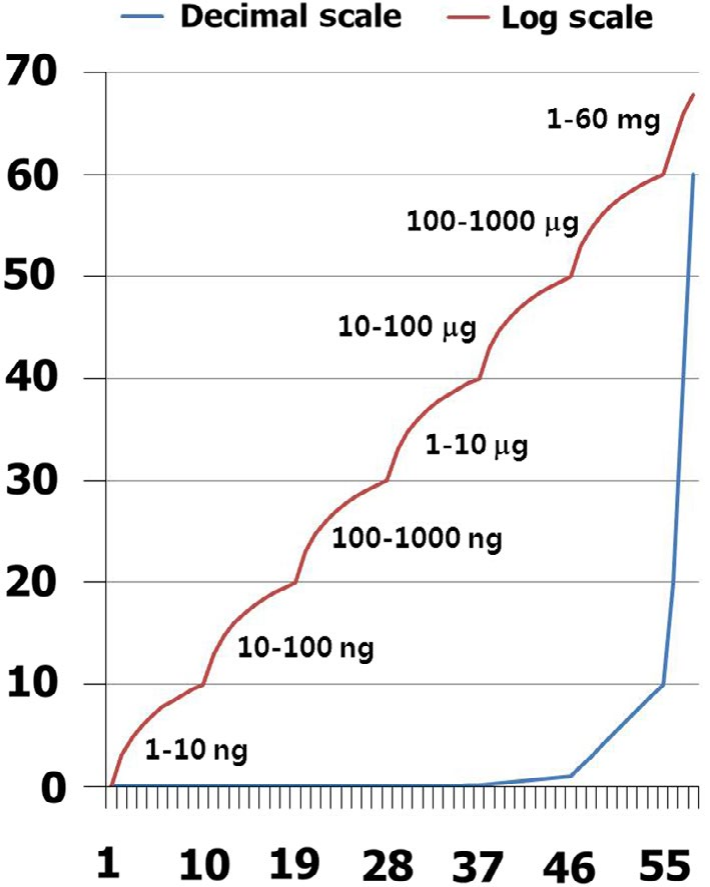
Incremental dosage curve. The incremental dose is every unit decimally within the basic dose. Dosage was increased 10‐fold according to the log scale

In contrast to the pre‐existing protocol for drug desensitization, the dosage escalation was modulated less steeply because severe anaphylactic shock was expected with steep dosage escalation. Desensitization using IFN‐gamma was initiated with the impediment dose. According to the theoretical background of anaphylactic food allergy, the incremental dose range was extremely low and rose steeply in the high‐dose range on a log scale (Table 1, Figure 2). Lidocaine, which did not contain any other additives, was dissolved in normal saline.

**Table 1:** CHH basic scheme for desensitization to intravenous lidocaine

Concentration	Progress									
1 ng/mL	1	2	3	4	5	6	7	8	9	10
10 ng/mL		2	3	4	5	6	7	8	9	10
100 ng/mL		2	3	4	5	6	7	8	9	10
1 mcg/mL		2	3	4	5	6	7	8	9	10
10 mcg/mL		2	3	4	5	6	7	8	9	10
100 mcg/mL		2	3	4	5	6	7	8	9	10
1 mg/mL										
10 mg/mL	1	2	3							

#### 2.5.2 Usage of IFN‐gamma

IFN‐gamma was administered for the desensitization of food allergies. However, in this case of intravenous lidocaine desensitization, IFN‐gamma was used only once per day. If impediment was faced during the desensitization, there was an attempt to overcome it the next day.

#### 2.5.3 Proceeding of desensitization and overcoming impediment using IFN‐gamma

The maximally severe clinical signs and symptoms were determined during the challenge test and in the early stage of desensitization as in the tolerance induction of food allergy. Minimal signs and symptoms which permitted progression to the next dose were determined during the challenge test and in the process of early desensitization.

An impediment is an allergy provocation by a certain dose during the desensitization; in the case of impediment, progression to the next step is impossible. In the previous protocols based upon previous basic concepts, the same dose was used repetitively with the expectation that the allergic reactions would diminish or disappear. However, in this case, the patient exhibited a more severe allergic reaction with the repetition of the same challenge dose; therefore, desensitization was deemed to have failed, or the challenging dose needed to be reduced.

IFN‐gamma was used only to overcome the impediment. IFN‐gamma was not used if the patient did not show significant allergic signs or symptoms at the previous dose.

#### 2.5.4 Determination of failure and when to proceed with desensitization using IFN‐gamma

The desensitization using IFN‐gamma was determined to be a failure when the patient exhibited persistent severe allergic signs and symptoms which the patients could not tolerate, or when more severe reactions by repeated challenge with same impediment dose occurred, even using IFN‐gamma. Desensitization was allowed to progress when the symptoms and signs were decreased with same impediment dose using IFN‐gamma.

#### 2.5.5 Baseline allergic responses as ignorable responses, untoward side effects of IFN‐gamma, pharmacologic effects of lidocaine

**Figure 3:**
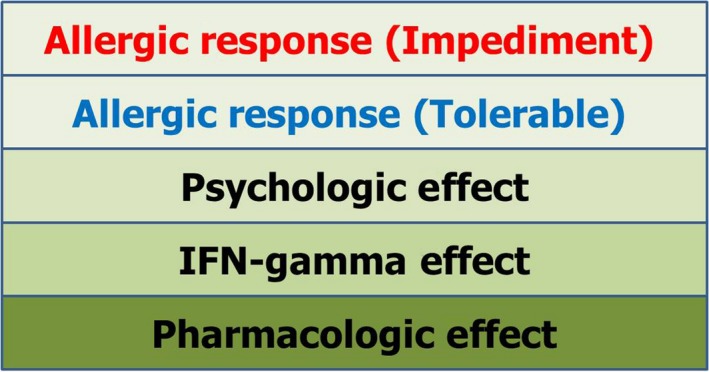
Clinical responses to intravenous challenge and desensitization. Allergic responses (impediment) are defined as responses that cannot be ignored. Allergic responses (tolerable) are defined as those responses that can be ignored, allowing for the desensitization to proceed. The psychological effects, side effects of IFN‐gamma and pharmacologic effects of treating allergenic drug should be considered during the drug desensitization

To determine impediment based upon the patient's signs and symptoms, ignorable responses should be defined as baseline symptoms and signs during challenge test and desensitization. In addition, untoward side effects of IFN‐gamma and pharmacologic effects of lidocaine, and psychologic effects due to psychologic trauma from a history of anaphylaxis should be differentiated (Figure 3). Only when the patient showed significant allergic responses was it deemed an impediment.

#### 2.5.6 Interval for the next challenge

Intervals for the challenge were determined by the disappearance of significant symptoms and signs that were provoked by previous challenges, considering the pharmacologic effects of lidocaine and the persistent allergic responses. However, when the signs and symptoms were strong, one more interval was prepared until the signs and symptoms reduced to zero or at least to a baseline.

#### 2.5.7 Application of IFN‐gamma

IFN‐gamma (Intermax gamma, LG Chemistry^®^, Seoul, Korea) was administered subcutaneously at a dose of 150 MU (37.5 µg) on the arm 15 minutes before the challenge of the impediment dose, as with anaphylactic food allergies. However, if the patient tolerated this dose well, the IFN‐gamma dosage was increased by 2 MU (50 µg) in the middle of treatment. Acetaminophen 650 mg was prescribed 15 minutes before the IFN‐gamma injection to avoid untoward side effects of IFN‐gamma, including headache, myalgia, abdominal pain, etc IFN‐gamma was administered early in the morning.

### 2.6 Intravenous challenge test and determination of the challenge interval

An intravenous challenge test was performed according to the protocol at day #1 of admission. A single vial of lidocaine contains 20 mL with a concentration of 2% and contains 36 mg. Lidocaine was diluted using normal saline for the skin prick test, intradermal test, and intravenous challenge. For the use of lidocaine in dental treatment, the target test dose was 36 mg with the assumption that a total vial of 2% lidocaine is used for local anesthesia and is absorbed directly into the circulation.

Lidocaine was challenged intravenously sequentially along the basic dosage protocol for the challenge test and desensitization, as shown in Table 1. The initial testing dose was 1 ng, and the dose was increased in increments of 1 ng within the range of 1‐10 ng and by 10 ng within the range of 10‐100 ng. The dose was increased 10‐fold in every step.

At a dose of 20 ng, the patient experienced a slight periorbital burning sensation and sneezing, which were suspected as allergic responses. With the injection of 30 ng, the patient again experienced burning sensations which were spread to the periorbital area and posterior neck through face along with sweating. When 30 ng of lidocaine was injected again, the patient exhibited more severe anaphylactic symptoms including immediate abdominal discomfort, vomiting, and respiratory difficulty. The diagnosis of anaphylactic lidocaine allergy was made concretely. The symptoms and signs disappeared after 1 hour, and the interval of challenge was determined to be 1 hour.

### 2.7 Failure of desensitization using a pre‐existing protocol

Desensitization was tried in a pre‐existing concept, and 30 ng of lidocaine was challenged as the third trial for the desensitization 1 hour after the diagnosis was made. However, the patient exhibited additional symptoms, such as laryngeal edematous, voice change, and a choking sensation. Lidocaine 30 ng was challenged three times, but the patient reacted more strongly with more signs and symptoms, and the clinical severity increased with the dose repetition. The patient received 30 ng of lidocaine three times intravenously, and the clinical reactions were aggravated by the repetition. It was predicted that anaphylactic shock would occur upon challenging with an increased dose or by continued repetition with same dose.

### 2.8 Desensitization to lidocaine using IFN‐gamma: Overcoming impediment using IFN‐gamma

**Figure 4:**
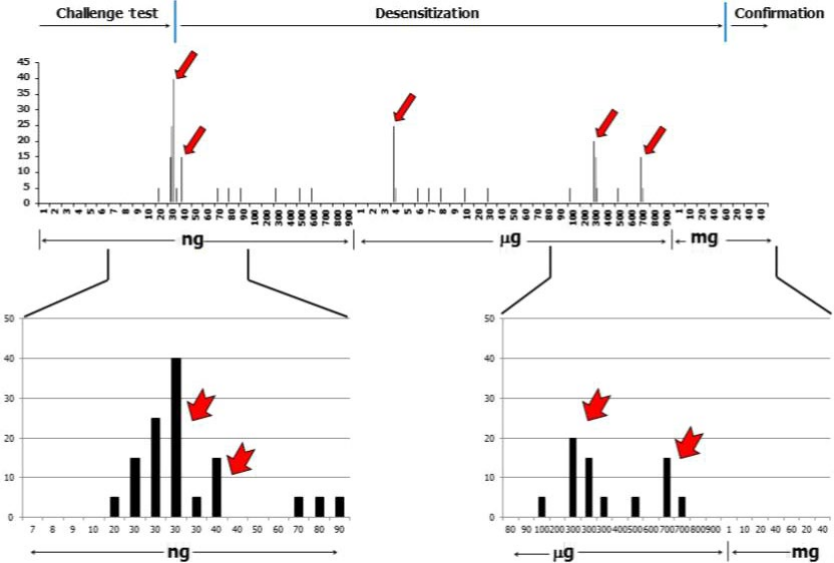
Progress of challenge and desensitization of lidocaine allergy. A, Overall desensitization progress. The red arrow indicates impediment and the administration of IFN‐gamma. The left vertical blue line indicates the border between the challenge test and desensitization, and the right vertical blue line indicates the border between desensitization and the post‐desensitization confirmative challenge to assure tolerance acquisition for intravenous lidocaine. B, Bridging between the challenge test and desensitization in the first impediment. The vertical red line indicates bridging from the challenge test and desensitization by administrating IFN‐gamma. C, Overcoming the impediment, and the confirmative challenge to assure tolerance acquisition for intravenous lidocaine

Desensitization using the pre‐existing concept failed or dosage modulation was needed. At this point, it was decided to introduce IFN‐gamma with the expectation that allergen‐specific tolerogenic effects would overcome the impediment at the dose of 30 ng (Figure 4). The starting dose for desensitization was determined as 30 ng as the minimum allergy‐provoking dose, and the target dose was 40 mg for dental care, which was deemed to be a non‐toxic dose for cardiovascular effects.

Desensitization using IFN‐gamma was restarted in the early morning of admission on day #2 with the dose of 30 ng, and the patient exhibited more severe allergic reactions with the repetition. Acetaminophen was administered orally 30 minutes before the challenge to prevent the untoward effects of IFN‐gamma, and IFN‐gamma was injected at a dose of 37.5 µg (150 million units) subcutaneously 15 minutes before the lidocaine challenge. Lidocaine 30 ng was challenged for desensitization, and, surprisingly, the signs and symptoms were dramatically decreased and included only a burning sensation of the periorbital area and posterior neck. With the repeated challenge of 30 ng, the patient experienced only the burning sensation of the periorbital area and posterior neck, which were considered baseline and ignorable signs and symptoms of allergic responses. After the IFN‐gamma injection, the patient reported feeling myalgia, which was regarded as a baseline sign and symptom resulting from IFN‐gamma during the desensitization process.

The patient was challenged with an increased dose of 40 ng and exhibited vomiting and laryngeal edema immediately; thus, the challenge was terminated. On admission day #3 at 6:30 am, the patient was pre‐treated with Tylenol 650 mg for the prevention of the untoward side effects of IFN‐gamma. IFN‐gamma was injected subcutaneously 15 minutes after the Tylenol intake. Lidocaine 40 ng was challenged 15 minutes after the IFN‐gamma injection as performed for injection 4. No allergic signs or symptoms appeared, except for myalgia, which was considered an effect of IFN‐gamma.

The desensitization proceeded, and the patient met impediment five times at the dose of 30, 40 ng, 4, 300, and 700 μg, which were overcome using IFN‐gamma. The target dose of 40 mg was reached on the admission day #8.

### 2.9 Confirmation of tolerance acquisition

After successfully reaching the target dose by desensitization, tolerance acquisition was confirmed by repeating the intravenous injection of lidocaine. One day after finishing the desensitization, 20 mg in the morning and 40 mg in the evening were injected without any symptoms or signs. Two days after finishing desensitization, 40 mg of lidocaine was injected, again without any symptoms and signs. The intradermal test for lidocaine was repeated, and the patient showed a negative result (Figure 1B).

Finally, 3 days after finishing desensitization, the patient received local anesthesia with lidocaine (27 mg) and safely underwent dental treatment (extraction of two teeth). One day after the dental treatment, patient was discharged.

## 3 DISCUSSION

In the case of anaphylactic shock, there is no time to think.^11^ Although extremely rare, allergy to lidocaine should be taken seriously in the presence of suggestive history.^12^ Fatal anaphylactic reaction to lidocaine was reported as early as 1957.^1^

### 3.1 Diagnosis of lidocaine allergy

Unfortunately, there is no reliable in vitro allergy diagnostic test available for routine use.^13^ Type I hypersensitivity to lidocaine has been confirmed by demonstrating specific IgE antibodies against the drug and was reported in relatively few cases.^5^, ^14^, ^15^, ^16^, ^17^ In this report, the skin prick test was negative, but the intradermal test was strongly positive. The skin prick test was performed with an undiluted concentration in this trial, as in the previous trial.^18^ Anaphylactic shock following the administration of lidocaine after a negative skin test has also been reported.^19^ Intradermal testing and subcutaneous challenge indicated that it may be safe to use lidocaine as an injectable local anesthetic in the future.^20^

### 3.2 Intravenous provocation test: minimal allergy provocation dose of lidocaine and incremental dose

Even from the skin prick test, systemic reactions have occurred.^21^ Thus, the intravenous challenge test was performed very cautiously because of the potential for fatal anaphylactic reactions. There was not sufficient information about the minimal allergy provocation dose of lidocaine. In this case, the intravenous challenge protocol was scheduled from an extremely low dose concerning the initial challenge dose because the sensitivity to lidocaine for allergy provocation is not well understood (Table 1, Figure 2).

In the intravenous challenge test, although the patient exhibited suspected allergic responses at a lidocaine dose of 20 ng, the minimal allergy provocation dose was 30 ng, at which symptoms and signs appeared. The patient fell into anaphylaxis after the intravenous injection of 5 mg of lidocaine,^17^ and there have been reports of using the usual or full therapeutic dose of intravenous lidocaine.^18^, ^22^ The precise minimal allergy provocation dose according to the intravenous provocation test was not described well, which may be because there was no established concept for desensitization at that time. The minimal provocation dose of intravenous lidocaine is very important to establish in designing the protocol for the challenge test and desensitization for intravenous lidocaine as well as other intravenous drugs.

The incremental dose was designed in a condensed manner compared to the incremental dose in specific oral tolerance induction for anaphylactic IgE‐mediated food allergy.^7^ At every range, the dosage range was increased by 10‐fold, as with the dosage protocol in specific oral tolerance induction for anaphylactic IgE‐mediated food allergy (Table 1, Figure 2).

### 3.3 Bridging between the challenge test and desensitization and the initial therapeutic dose

In the case of anaphylactic IgE‐mediated food allergy, the initial therapeutic dose was 1/10 of the minimal provocation dose.^23^ However, in the present trial, the initial therapeutic dose was determined as the minimal allergy provocation dose as the concept to overcome the impediment using IFN‐gamma.

### 3.4 Baseline score

From the information gained from the challenge test, slight symptoms and signs below a score of 5 were regarded as baseline symptoms, and signs such as a slight burning sensation or myalgia appeared after the intravenous administration of 20 ng of lidocaine.

### 3.5 Necessity of IFN‐gamma as a new therapeutic concept for drug desensitization

Factors to consider regarding the desensitization to drugs include the time required and the success rates of desensitization. Moreover, a past limitation of drug desensitization was that, in patients who failed the desensitization protocol, a new attempt to desensitize was not undertaken.^6^

**Figure 5:**
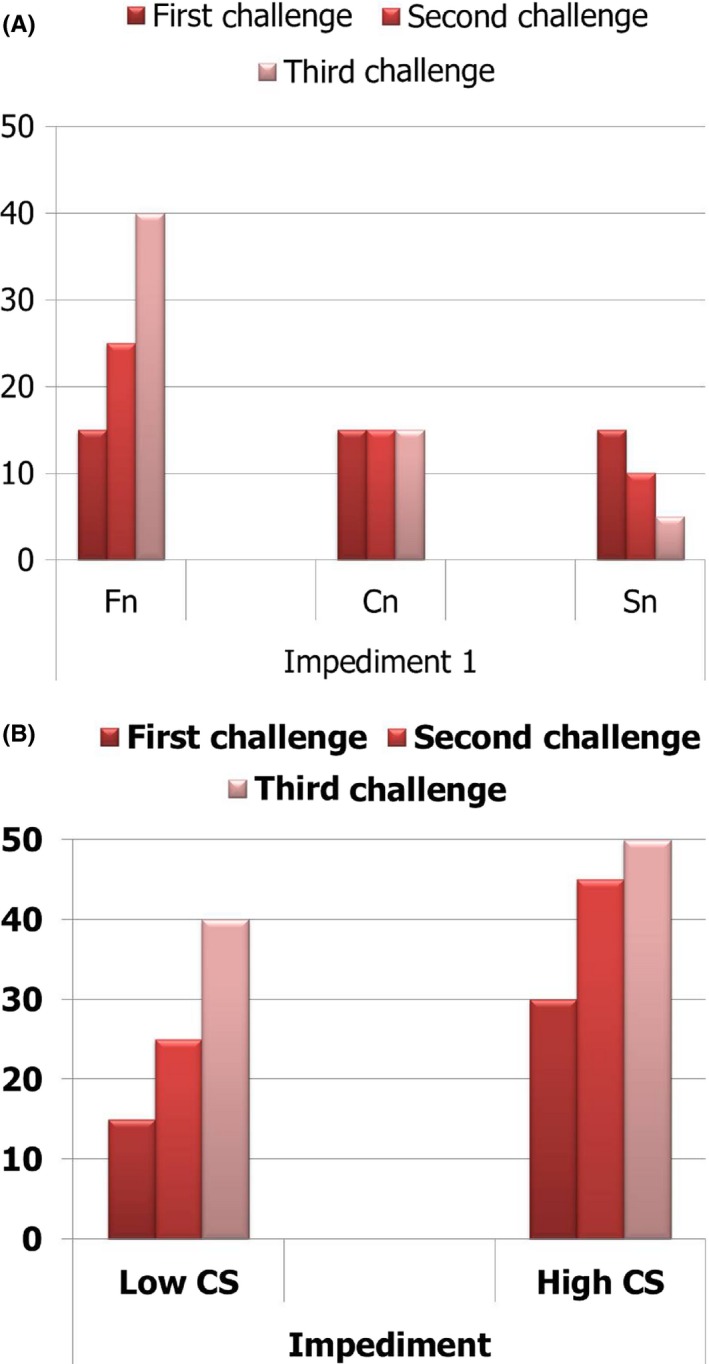
Decision of impediment cycle. A, Fn, Failed point of proceeding with the desensitization (Impediment), at which point the desensitization cannot proceed with conventional desensitization concept; Cn, Considerable point of proceeding of desensitization, at which point the desensitization can be proceeded by dosage modulation; Sn, Successful point of proceeding of desensitization. B, Low CS, impediment at the extremely low dose, High CS, impediment at the high dose. When impediment is met at the extremely low dose, the patient can be considered to be very sensitive, and it is very difficult to modulate the challenging dose. However, when impediment is met at the high dose, the dosage reduction can be considered more conveniently. IFN‐gamma can be considered when impediment is met at Fn in which the dosage is extremely low

The first impediment is the critical point at which to decide to introduce IFN‐gamma in the lidocaine desensitization. At the first impediment, the allergic response by the first challenge was not severe but was aggravated by repetition (Figures 4 and 5A). If the allergic responses are severe (Figure 5B), a re‐challenge with a reduced dose (relative to the first impediment dose (30 ng)) may be considered, but 30 ng is a very low dose, and the prior dose is 20 ng. The dosage range between 20 and 30 ng is too narrow to be modulated, and the dosage modulation was regarded as the meaningless. Conclusively, it was decided that desensitization with the previous protocol possibly failed, and IFN‐gamma was considered to be introduced for desensitization.

IFN‐gamma was reported to be effective in atopic dermatitis,^24^, ^25^, ^26^ and the proper use of IFN‐gamma was suggested.^27^ IFN‐gamma was used for allergen‐specific tolerance induction in desensitization for house dust mites^28^ and for non‐IgE‐mediated food allergy in atopic dermatitis.^8^ As a next step, IFN‐gamma was tried successfully in anaphylactic Ig‐mediated food allergy.^29^ From these reports, IFN‐gamma was revealed to have tolerogenic effects for both inhalant and food allergens. The clinical protocol for tolerance induction for food allergies using IFN‐gamma is well‐established,^7^ and the mechanisms for tolerance induction for allergen‐specific allergies were studied as the induction of allergen‐specific regulatory B cells in vivo and in vitro.^30^, ^31^

**Figure 6:**
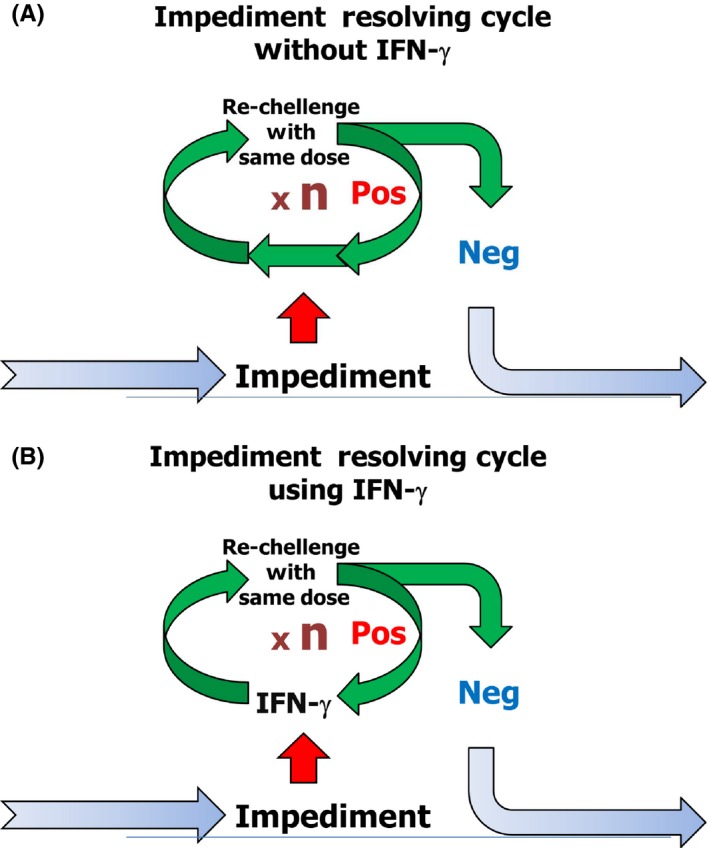
Impediment resolution cycle with/without IFN‐gamma. If impediment is met, desensitization enters the impediment resolving cycle. When the impediment was not resolved with conventional concept (A), IFN‐gamma was introduced as pretreatment, and the drug was challenged with the impediment dose repetitively until the allergic reactions disappeared (B)

IFN‐gamma was introduced in this trial for intravenous lidocaine desensitization. The principle of overcoming a prior dose^29^ in the tolerance induction for anaphylactic IgE‐mediated food allergy using IFN‐gamma was applied based upon the same concept as that used for the impediment resolution cycle using IFN‐gamma in drug desensitization (Figure 6). If patients exhibited allergic reactions to a certain dose (n), the desensitization process entered the impediment resolution cycle repetitively, in which IFN‐gamma was used for pretreatment, and the allergy provocation dose (n) was challenged repetitively until the patient exhibited allergic responses similar to those exhibited with the allergy provocation dose (n), and no more. Then, the patient proceeded to the next dose, sequentially, according to the dose protocol.

**Figure 7:**
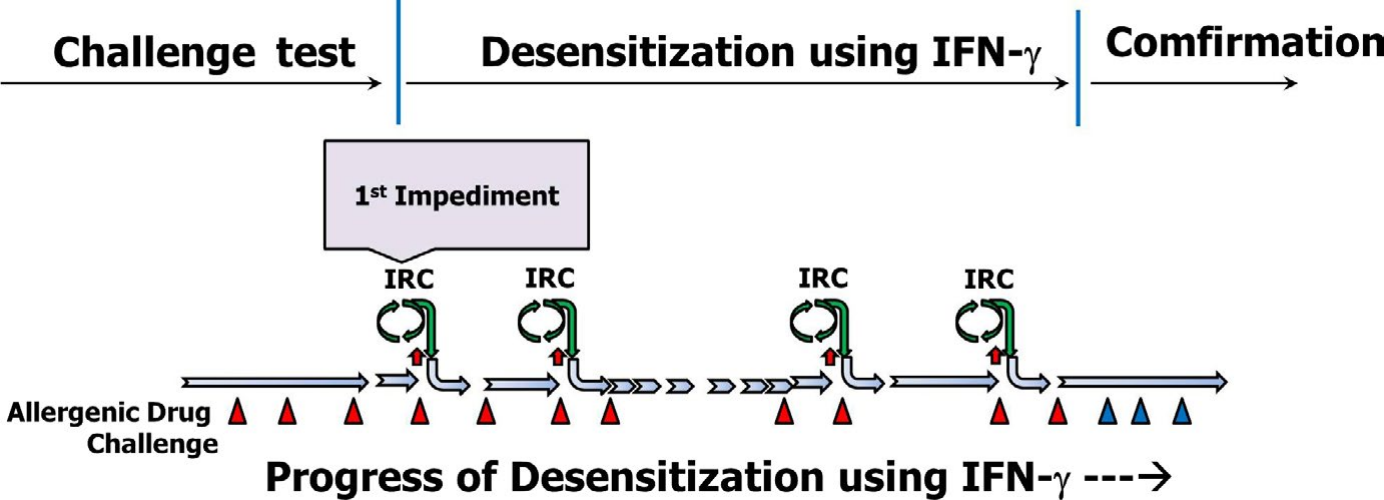
Scheme representing the progress of lidocaine desensitization using IFN‐gamma. First, the challenge test was performed. The minimal allergenic dosage was decided and used as the first treatment dose. If impediments are met during the desensitization, the process enters the impediment resolution cycle. If impediment is resolved, then desensitization is allowed to progress to a further dosage of the allergenic drug. (IRC, impediment resolving cycle; Red triangle, Positive challenge with allergenic state; Blue triangle, Negative challenge with tolerable state)

In this case, an impediment appeared five times during the desensitization and each was resolved successfully (Figure 7). Interestingly, impediments were met at the low‐dose range (30 and 40 ng) with relatively severe reactions and also at the high‐dose range with relatively weak reactions (Figure 4).

However, the application of IFN‐gamma for drug allergies was different from that for food allergies. In the case of food allergies, IFN‐gamma was administered for every challenge with the interval of at least one day.^7^, ^8^, ^23^, ^29^ In the case of drug desensitization, if the patient met the impediment, IFN‐gamma was given, and the desensitization proceeded until the patient met the next impediment. IFN‐gamma was not used when the patient did not exhibit an allergic reaction to the prior dose. IFN‐gamma was used only when impediment was met and successfully overcome by challenging with the allergy‐provoking dose one or two times. The use of IFN‐gamma for drug desensitization or tolerance induction of food allergies is a great advance. Not only does IFN‐gamma appear to have allergen‐specific tolerogenic effects for drug allergies but also the allergen‐specific tolerogenic effects of IFN‐gamma were proven more concretely. As with an anaphylactic food allergy, the minimal allergy provocation dose was as low as 30 ng for the lidocaine allergy.^7^, ^29^

In contrast to food allergies, the pharmacologic effects and toxic effects of lidocaine should be considered to determine the desensitization protocol for lidocaine due to its cardiologic effects and other toxic effects as a drug. Additionally, the patient should be admitted for intravenous desensitization, and this situation should be reflected in the desensitization protocol. The protocol was set as rush immunotherapy, and the bolus dose and accumulated dose of lidocaine during desensitization should be determined based upon consultations with a cardiologist and a pharmacist. In addition, during the intravenous lidocaine desensitization, not only pharmacologic and toxic effects of lidocaine but also untoward side effects of IFN‐gamma and psychologic effects should be discriminated from the allergic reactions.

From this report, desensitization to lidocaine using IFN‐gamma was successful after a failed desensitization case. Desensitization using IFN‐gamma for lidocaine is a comprehensive, state of the art method of tolerance induction for drug allergies from experience with tolerance induction for food allergies. Advances were achieved for more precise treatment for tolerance induction for drug allergy and food allergy along with results from food allergy and drug allergy trials.

Active desensitization using IFN‐gamma was suggested rather than avoiding desensitization or using passive desensitization. IFN‐gamma appears to be tolerogenic for drug allergies as with food allergies. IFN‐gamma may also be applicable for the desensitization to many other drugs, and more investigation may be needed.

## CONFLICT OF INTEREST

None declared.

## AUTHOR CONTRIBUTIONS

GN: is a main author for this manuscript; CKP: the responsible doctor for the treatment during this study; and CWH: discussed about theoretical background during the treatment in this study.
